# Formulation of Time-Fractional Electrodynamics Based on Riemann-Silberstein Vector

**DOI:** 10.3390/e23080987

**Published:** 2021-07-30

**Authors:** Tomasz P. Stefański, Jacek Gulgowski

**Affiliations:** 1 The Faculty of Electronics, Telecommunications and Informatics, Gdansk University of Technology, 80-233 Gdansk, Poland; tomasz.stefanski@pg.edu.pl; 2 The Faculty of Mathematics, Physics and Informatics, University of Gdansk, 80-308 Gdansk, Poland

**Keywords:** Maxwell’s equations, fractional derivatives, Riemann-Silberstein vector

## Abstract

In this paper, the formulation of time-fractional (TF) electrodynamics is derived based on the Riemann-Silberstein (RS) vector. With the use of this vector and fractional-order derivatives, one can write TF Maxwell’s equations in a compact form, which allows for modelling of energy dissipation and dynamics of electromagnetic systems with memory. Therefore, we formulate TF Maxwell’s equations using the RS vector and analyse their properties from the point of view of classical electrodynamics, i.e., energy and momentum conservation, reciprocity, causality. Afterwards, we derive classical solutions for wave-propagation problems, assuming helical, spherical, and cylindrical symmetries of solutions. The results are supported by numerical simulations and their analysis. Discussion of relations between the TF Schrödinger equation and TF electrodynamics is included as well.

## 1. Introduction

Recently, the problem of electrodynamics of fractional order (FO) has been introduced in the literature. Between various approaches, one can notice the generalizations of Maxwell’s equations involving spatial and temporal FO derivatives [1,2,3,4,5], only spatial derivatives [6,7,8], and only temporal FO derivatives [9,10,11,12]. Such generalizations of Maxwell’s equations employ FO derivatives in order to describe the dynamics of electromagnetic systems with memory and energy dissipation [13]. Furthermore, the use of FO derivatives in electrodynamics allows one to define the electric potential based on the concept of FO poles [14,15,16]. Hence, a concept of the fractional multipole is formulated, which allows for calculating the electric potential at the distance *r* from the source, which is proportional to $r^{-\alpha }$ (where α∈R is not integer). This approach can be applied to solve some electrostatic-image problems, involving perfectly conducting wedges and cones. In [6], the fractionalization of the duality principle in electrodynamics is proposed, making use of the FO curl operator. This approach can be applied to solve some reflection [17,18] and diffraction [19] problems. Afterwards, in [20], the FO curl operator is proven to be useful for the analysis of the transmission of electromagnetic waves through a slab of reciprocal chiral medium. Hence, the FO curl operator, to some extent, appears to be mathematically equivalent to a chiral medium. As a consequence, some electromagnetic characteristics of media can be modelled using the FO derivatives.

In this paper, we extend the formulation of time-fractional (TF) electrodynamics with the use of the Riemann-Silberstein (RS) vector, i.e., the complex vector which combines simultaneously the electric and magnetic field. This vector was initially introduced by Bernhard Riemann in the lectures on partial-differential equations, which were edited and published in 1901 by Weber [21]. Then, Silberstein studied the RS vector [22,23] and employed it for the quaternionic formulation of the relativity theory [24]. For the historical background of the RS vector, one is referred to [25]. In [25,26,27,28], it is demonstrated that the RS vector provides a uniform and elegant description of electromagnetism, which connects classical and quantum electrodynamics. In [29,30], it is demonstrated that the RS vector is useful for the analysis of antenna and propagation problems in engineering.

To the best of Authors’ knowledge, none of formulations of FO electrodynamics has been extended yet using the RS vector. Furthermore, the existing formulations of electrodynamics based on the RS vector do not include the energy dissipation, i.e., they are limited to a homogeneous and static medium, which is usually a vacuum. However, the time-domain fractionalization of Maxwell’s equations allows for inclusion of energy dissipation, which is the intrinsic property of media described by fractional-order models (FOMs). Furthermore, it is possible to demonstrate that the energy density of the electromagnetic field and media described by FOM, which is spent to create the field and change the momentum of medium, reflects the entire previous history of the process. Hence, the application of FOM allows for description of memory effects in electrodynamics. Therefore, we investigate this topic focusing on classical-physics solutions. Then, we can demonstrate relations between the TF Schrödinger equation and TF electrodynamics using the proposed formulation based on the RS vector.

## 2. Preliminaries

In this section, we introduce the basic notation and terminology, which is used throughout the paper.

### 2.1. Basic Notations

The standard engineering notation for the imaginary unit $j=\sqrt{-1}$ is used throughout the paper. We denote real and imaginary parts of the complex number *s* by ℜ(s) and ℑ(s), respectively. Then, the conjugate to the complex number *s* is denoted as $s^{*}$.

The Fourier transformation is defined for the absolutely integrable function of time f:R→C (i.e., f(t)) by the formula

(1) $$F\left(f\right)\left(\omega \right)=F\left(\omega \right)=\int_{-\infty }^{+\infty }e^{-j\omega t}f\left(t\right)dt$$

whilst the inverse Fourier transformation is given by the formula

(2) $$F^{-1}\left(F\right)\left(t\right)=f\left(t\right)=\frac{1}{2\pi }\int_{-\infty }^{+\infty }e^{j\omega t}F\left(\omega \right)d\omega .$$

For the absolutely integrable function of space $g:R^{3}\to C$ (i.e., g(r)), we define the spatial Fourier transformation by the formula

(3) $$F\left(g\right)\left(k\right)=G\left(k\right)=\int_{R^{3}}e^{jk\cdot r}g\left(r\right)d^{3}r$$

whilst the inverse spatial Fourier transformation is given by the formula

(4) $$F^{-1}\left(G\right)\left(r\right)=g\left(r\right)=\frac{1}{{\left(2\pi \right)}^{3}}\int_{R^{3}}e^{-jk\cdot r}G\left(k\right)d^{3}k.$$

The convention of the Fourier transformation can be changed into to the one applied in physics and mathematics. For this purpose, the imaginary unit *j* should be replaced by −i (where $i=\sqrt{-1}$) in (1)–(4).

Throughout the paper, a branch of logarithm with the domain covering all purely imaginary numbers of the form jω is taken, where ω∈R\{0}

(5) $$\ln\left(s\right)=\ln(|s|e^{j\theta })=\ln|s|+j\theta$$

for $s=\left|s\right|e^{j\theta }$, s≠0 and θ∈(−π,π). From (5), one obtains

(6) $$\ln\left(j\omega \right)=\ln|\omega |+j\frac{\pi }{2}\mathrm{sgn}\left(\omega \right).$$

Then, we define complex-valued function $s\mapsto s^{\nu }$ for ν∈R by the selection of an appropriate branch of complex logarithm

(7) $$s^{\nu }=e^{\nu \ln s}=e^{\nu \ln|s|+j\nu \theta }={\left|s\right|}^{\nu }e^{j\nu \theta }$$

for $s=\left|s\right|e^{j\theta }$, s≠0 and θ∈(−π,π). One can notice that the function $s\mapsto s^{\nu }$ is holomorphic for $s\in C_{+}$, where $C_{+}=\left\{s\in C:ℜ\left(s\right)>0\right\}$. Let us consider as a special case the function G:R\{0}→C given by

(8) $$G\left(\omega \right)={\left(j\omega \right)}^{\nu }=e^{\nu \ln(j\omega )}={\left|\omega \right|}^{\nu }e^{j\nu \frac{\pi }{2}\mathrm{sgn}\left(\omega \right)}.$$

Hence, one obtains

(9) $$ℜ{\left(j\omega \right)}^{\nu }={\left|\omega \right|}^{\nu }\cos\left(\nu \frac{\pi }{2}\right)\;\mathrm{and}\;ℑ{\left(j\omega \right)}^{\nu }={\left|\omega \right|}^{\nu }\mathrm{sgn}\left(\omega \right)\sin\left(\nu \frac{\pi }{2}\right)$$

for ω∈R\{0}.

### 2.2. FO Calculus

The Marchaud derivative of the order β∈(0,1) is defined as

(10) $$D^{\beta }f\left(t\right)=\frac{\beta }{\Gamma (1-\beta )}\int_{0}^{+\infty }\frac{f(t)-f(t-\tau )}{\tau^{1+\beta }}d\tau =$$

(11) $$=\frac{\beta }{\Gamma (1-\beta )}\int_{-\infty }^{t}\frac{f(t)-f(\tau )}{{\left(t-\tau \right)}^{1+\beta }}d\tau .$$

Although the Marchaud derivative can be formulated for higher orders β>1 (see Section 5.6 in [31]), we do not refer to this more general case in our investigations. In (10) and (11), the function *f* is assumed to be smooth enough, e.g., $f\in C^{1}\left(R\right)$ with |f| bounded by some function not growing too quickly in ±∞ (more about these assumptions later). Applicability of the Marchaud derivative in our investigations stems from its advantages, refer to [32,33]:

- The Marchaud derivative of the order β∈(0,1) of the function *f* exists, if $f\in C^{1}\left(R\right)$ and $f^{\prime }\left(t\right)={O(|t|}^{\beta -1-\varepsilon })$ as |t|→+∞ for some ε>0 (see Section 5.4 in [31]). If additionally $f\in L^{p}\left(R\right)$ for p∈[1,1/β), then the Marchaud derivative coincides with the Riemann-Liouville derivative with a base point a=−∞.
- For the derivative of the order α of the exponent $e^{st}$ (where s∈C is fixed), one obtains
  (12) $$D^{\alpha }e^{st}=s^{\alpha }e^{st}$$
  for ℜ(s)≥0. As discussed in [34] (see (5) therein), this limitation stems from the memory of derivative, which covers the entire interval (−∞,t). Therefore, allowing for ℜ(s)<0 would lead to divergent integrals. In practical terms, it is not a limitation, because one should consider functions bounded at −∞, or even causal (i.e., equal to zero for arguments in (−∞,0).
- The Marchaud derivative, the Riemann-Liouville derivative with a base point in −∞ and the Grünwald-Letnikov derivative [31,35] are equivalent for a very broad class of functions.
- When the Marchaud derivative coincides with the Grünwald-Letnikov derivative for the function *f*, then one obtains
  (13) $$D^{\alpha }\left(D^{\beta }f\right)=D^{\beta }\left(D^{\alpha }f\right)=D^{\alpha +\beta }f$$
  where α,β>0.
- In some cases, we would like to assume not only that the Marchaud derivative of the function f:R→R exists, but also that this derivative belongs to an appropriate space $L^{p}\left(R\right)$. It is handled by Theorems 6.1 and 6.5 in [31]. The condition that guarantees that $D_{t}^{\beta }\left(f\right)=\phi \in L^{p}\left(R\right)$ is that $f\in H^{\lambda }\left(R\right)$ and $\lim_{t\to +\infty }f\left(t\right)=0$ for λ>max{β,1/p−β}. The function space $H^{\lambda }\left(R\right)$ denotes the space of Hölder continuous functions with appropriate behaviour in ±∞ (see (1.5) and (1.6) in [31]). One can check that, in order to belong to $H^{\lambda }\left(R\right)$, it is enough to assume that $f\in \;C^{1}\left(R\right)$ and
  (14) $$\int_{|t|\geq 1}|f^{\prime }{\left(t\right)|}^{q}{\left|t\right|}^{2(q-1)}dt<+\infty$$
  for $q\geq \frac{1}{1-\lambda }$. The last condition is satisfied, e.g., when $f^{\prime }\left(t\right)={O(|t|}^{-\lambda -1-\varepsilon })$ as |t|→+∞.
- We employ in our considerations some properties of the null space of the Marchaud derivative. We are not aware of any general result in this area, but we can show some reasonable behaviour assuming that ϕ is a Hölder continuous function. That is, if one assumes that $\phi \in H^{\lambda }\left(R\right)$ with exponent λ∈(β,1) and $\lim_{t\to \pm \infty }\phi \left(t\right)=0$, then $D^{\alpha }\phi =0$ implies ϕ=0. This is a consequence of Theorems 6.1 and 6.5 from [31].
- The appropriate Fourier transformation identity can be formulated for the Marchaud derivative (see formula (7.4) in [31])
  (15) $$F\left\{D^{\alpha }f\right\}={\left(j\omega \right)}^{\alpha }F\left\{f\right\}$$
  when $f\in C^{n}\left(R\right)$, α∈(n−1,n), and *f* along with derivatives up to the order *n* belongs to $L^{1}\left(R\right)$.

Due to these properties, the Marchaud derivative is preferable in our investigations focused on electrodynamics. It occurs that popular definitions of FO derivative, e.g., Riemann-Liouville and Caputo [31,35], do not satisfy the properties (12) and (13) in general, which are necessary for the analysis of the wave propagation in the media described by FOM (refer to [32,33]).

### 2.3. Vector Fields

It is necessary to formulate precise assumptions related to functions (i.e., vector fields), which we use in our considerations. Suppose now that $V\subset R^{3}$ is a compact volume with the boundary *S* being the piecewise smooth surface. For the vector field $X:V\times R\to R^{3}$ (or $X:V\times R\to C^{3}$), we always assume that this is $C^{2}$ map in the entire domain and the functions $X_{i}\left(x,y,z,\cdot \right)$ (i∈{x,y,z}) belong to $L^{1}\left(R\right)$ for all (x,y,z)∈V. Hence, the Fourier transformation (1) can be calculated with respect to the time variable

(16) $$\tilde{X}\left(x,y,z,\omega \right)=F\left\{X\left(x,y,z,\cdot \right)\right\}\left(\omega \right)$$

for a fixed (x,y,z)∈V. We also assume that the limits as |t|→+∞ of functions $X_{i}\left(x,y,z,\cdot \right)$ (i∈{x,y,z}) equal to zero and the time derivative $D_{t}^{1}X_{i}\left(x,y,z,\cdot \right)=O\left(t^{-c}\right)$ as |t|→+∞ for c>0 big enough. It implies that the derivative $D_{t}^{\beta }X\left(x,y,z,\cdot \right)\in L^{1}\left(R\right)$. One should note that having two functions f,g:R→R, satisfying the assumptions given above for which $D_{t}^{1-\beta }f=D_{t}^{1-\beta }g$, one obtains f=g.

Hence, the vector fields $E,H:V\times R\to R^{3}$ are given for which $D_{t}^{\beta }E,D_{t}^{\beta }H\in L^{1}\left(R\right)$. It means that the functions $\bar{E},\bar{H}:V\times R\to R^{3}$ can be defined such as

(17) $$\bar{E}\left(x,y,z,t\right)=\int_{-\infty }^{t}D_{t}^{\beta }E\left(x,y,z,\tau \right)d\tau =I_{t}^{1}D_{t}^{\beta }E\left(x,y,z,t\right)=I_{t}^{1-\beta }E\left(x,y,z,t\right)$$

(18) $$\bar{H}\left(x,y,z,t\right)=\int_{-\infty }^{t}D_{t}^{\beta }H\left(x,y,z,\tau \right)d\tau =I_{t}^{1}D_{t}^{\beta }H\left(x,y,z,t\right)=I_{t}^{1-\beta }H\left(x,y,z,t\right)$$

which are well defined and differentiable. In (17) and (18), $I_{t}^{1}$ denotes the integral of upper integration limit $\int_{-\infty }^{t}\left(\cdot \right)\;d\tau$ and $I_{t}^{1-\beta }=I_{t}^{1}D_{t}^{\beta }$. Hence, one obtains

(19) $$D_{t}^{1-\beta }\bar{E}\left(x,y,z,t\right)=E\left(x,y,z,t\right)$$

(20) $$D_{t}^{1-\beta }\bar{H}\left(x,y,z,t\right)=H\left(x,y,z,t\right).$$

In what follows, we often refer to the dot-product notation. For complex vectors $A,B\in C^{3}$, the product does not refer to the standard dot product over the field of complex numbers but to the bilinear form ‘borrowed’ from the real-valued case, i.e.,

(21) $$A\cdot B=A_{x}B_{x}+A_{y}B_{y}+A_{z}B_{z}.$$

Therefore, to be formal, when we want to refer to the standard dot product of complex vectors $A,B\in C^{3}$, we write:

(22) $$A\cdot B^{*}=A_{x}B_{x}^{*}+A_{y}B_{y}^{*}+A_{z}B_{z}^{*}.$$

## 3. RS Vector

In this section, we propose TF formulation of electrodynamics based on the RS vector. Our FO approach is consistent with the RS vector formulation of classical electrodynamics proposed in [25,29].

Let us consider symmetric Maxwell’s equations in free space

(23) $$\nabla \cdot D=\rho_{e}$$

(24) $$\nabla \times E=-\frac{\partial B}{\partial t}-M$$

(25) $$\nabla \cdot B=\rho_{m}$$

(26) $$\nabla \times H=\frac{\partial D}{\partial t}+J$$

where E and H are, respectively, the electric- and magnetic-field intensities, D and B are, respectively, the electric- and magnetic-flux densities, J and M are, respectively, the electric- and magnetic-current densities, and $\rho_{e}$ and $\rho_{m}$ are, respectively, the electric- and magnetic-charge densities. We assume the constitutive relations for the medium described by FOM in the following form [34,36]:

(27) $$\epsilon_{\beta }E=D_{t}^{1-\beta }D,\;0<\beta \leq 1$$

(28) $$\mu_{\gamma }H=D_{t}^{1-\gamma }B,\;0<\gamma \leq 1.$$

In (27) and (28), the parameters $\epsilon_{\beta }$ and $\mu_{\gamma }$ denote, respectively, FO equivalents of permittivity and permeability, whose SI units are as follows: $\left[\epsilon_{\beta }\right]=\frac{F}{s^{1-\beta }m}$, $\left[\mu_{\gamma }\right]=\frac{H}{s^{1-\gamma }m}$. In [37], the constitutive relation (27) is described as an ‘engineering’ model of dielectrics. Such a model describes memory effects and hereditary properties of electromagnetic field in media. It results not only from experimental results of Westerlund and Ekstam published in 1994, but also from the purely empiric Curie’s law formulated in 1889 [38]. Assuming analogous form of the constitutive relation for the magnetic field, one can write (28). When β=1 and γ=1, the constitutive relations for the linear medium, described by integer-order (IO) model (IOM) with the permittivity $\epsilon_{1}$ and the permeability $\mu_{1}$, are obtained

(29) $$D=\epsilon_{1}E$$

(30) $$B=\mu_{1}H.$$

For a vacuum, we assume that the permittivity and permeability are respectively denoted by $\epsilon_{0}$ and $\mu_{0}$. Comparing to the settings considered before [34], we assume the same FO of all the time derivatives in (23)–(26) in order to formulate TF Maxwell’s equations based on the RS vector. In our considerations, we assume that $\frac{1}{2}\leq \beta =\gamma \leq 1$. Therefore, one can write (23)–(26) as

(31) $$\nabla \cdot D=\rho_{e}$$

(32) $$\nabla \times E=-\mu_{\beta }D_{t}^{\beta }H-M$$

(33) $$\nabla \cdot B=\rho_{m}$$

(34) $$\nabla \times H=\epsilon_{\beta }D_{t}^{\beta }E+J.$$

Let us now define the parameter

(35) $$Z_{f}=\sqrt{\frac{\mu_{\beta }}{\epsilon_{\beta }}}$$

whose the SI unit is Ohm, so may be considered as a form of the wave impedance. Let us formulate the RS vector as follows:

(36) $$F=\frac{1}{\sqrt{2}}\left(\frac{E}{\sqrt{Z_{f}}}+jH\sqrt{Z_{f}}\right).$$

Our definition of the RS vector is consistent with the definition applied in engineering [29,30], where the E, H vectors are usually employed to describe electromagnetic systems (e.g., antennas and waveguides). In order to write Maxwell’s equations for the RS vector F, we need to introduce an additional notation. Let us define

(37) $$c_{\beta }=\frac{1}{\sqrt{\mu_{\beta }\epsilon_{\beta }}}$$

(38) $$K=-\frac{1}{\sqrt{2}}\left(\frac{M}{\sqrt{Z_{f}}}-jJ\sqrt{Z_{f}}\right)$$

(39) $$\rho =\frac{1}{\sqrt{2}}\left(\rho_{e}\sqrt{Z_{f}}+j\frac{\rho_{m}}{\sqrt{Z_{f}}}\right).$$

For a vacuum when β=1, one obtains $c_{1}=c={\left(\mu_{0}\epsilon_{0}\right)}^{-\frac{1}{2}}$. With the definitions given above, FO Maxwell’s Equations (31)–(34) can be written as

(40) $$\nabla \times F=jc_{\beta }^{-1}D_{t}^{\beta }F+K$$

(41) $$\nabla \cdot F=c_{\beta }D_{t}^{1-\beta }\rho .$$

Without sources (K=0), (40) can be written as the evolution equation analogous to the TF Schrödinger equation [39,40,41] with the Hamiltonian $\hat{H}=c_{\beta }\left(p\cdot \Sigma \right)$, i.e.,

(42) $$c_{\beta }\left(p\cdot \Sigma \right)F=j\hbar D_{t}^{\beta }F$$

where p=−jℏ∇ and Σ denotes the spin-1 counterparts of the Pauli matrices

$$\Sigma_{x}=\left[\begin{array}{ccc} 0 & 0 & 0\\ 0 & 0 & -j\\ 0 & j & 0 \end{array}\right],\Sigma_{y}=\left[\begin{array}{ccc} 0 & 0 & j\\ 0 & 0 & 0\\ -j & 0 & 0 \end{array}\right],\Sigma_{z}=\left[\begin{array}{ccc} 0 & -j & 0\\ j & 0 & 0\\ 0 & 0 & 0 \end{array}\right].$$

The TF Schrödinger equation $\hat{H}\psi =j\hbar D_{t}^{\beta }\psi$ differs from the ordinary Schrödinger equation due to the TF derivative of the wave function ψ. The ordinary Schrödinger equation $\hat{H}\psi =j\hbar \frac{\partial }{\partial t}\psi$ has a mathematical form of a diffusion equation and can be obtained for a quantum-mechanical particle by considering a Gaussian-probability distribution in the space of all possible paths [39,42]. Hence, one can obtain various types of Schrödinger equations for non-Gaussian distributions. For instance, Feynman’s path-integral approach is employed in [43] to construct space-fractional quantum mechanics. That is, the Schrödinger equation is obtained with FO space derivatives due to the application of Levy distributions instead of Gaussian distributions to the set of possible paths.

Similarly, the TF Schrödinger equation is obtained if one considers non-Markovian evolution [39,40]. Analogously, the TF Maxwell’s Equations (31)–(34), which can be formulated in the form similar to the TF Schrödinger equation, actually stem from the application of non-standard memory function in a transport equation in order to include a temporal dispersion [9].

In general, the FO derivatives stem from relaxation and oscillation processes that exhibit memory and delay. For instance, the FO derivative in the oscillator equation is equivalent, to some extent, to the presence of a dissipative term in the standard oscillator equation [44]. However, this damping represents an intrinsic feature of the motion equation and does not result from additional dissipation like for a damped harmonic oscillator. Similar dumping of dynamics is also visible for the harmonic oscillator described by the TF Schrödinger equation [41]. Analogously, as it is shown later, the application of non-local formalism of FO derivatives to describe complex electromagnetic systems with memory also provides the energy dissipation, which is the intrinsic property of such systems.

The TF Schrödinger equation describes a non-relativistic particle [40]. Analogously, the application of FO constitutive relations (27) and (28) to Maxwell’s equations provides non-relativistic solutions obtained for the diffusion-wave equation [45,46,47]. This equation delivers solutions classified as intermidiate cases between the diffusion and the wave processes, which are different in terms of response to a localized disturbance. That is, the diffusion equation models a disturbance which spreads infinitely fast, whereas the wave equation models a disturbance with constant propagation velocity. Hence, for the TF diffusion-wave equation, the fundamental solution possesses a maximum that spreads with a finite velocity and the propagation velocity of a disturbance is infinite [45]. To sum up, the TF diffusion-wave equation is non-relativistic similarly to the standard diffusion equation.

Using the definition of the RS vector (36), one can find that Poynting’s vector [48,49]

(43) S=E×H

can be written as

(44) $$S=jF\times F^{*}.$$

Then, one can find that

(45) $${\left|F\right|}^{2}=F\cdot F^{*}=\frac{1}{2}\left(\frac{{\left|E\right|}^{2}}{Z_{f}}+{\left|H\right|}^{2}Z_{f}\right).$$

It has the same unit as the Poynting vector, i.e., $[F\cdot F^{*}]=$ Watt/m${}^{2}$ and can be interpreted as the power density of the plane wave propagating in a medium whose the wave impedance is $Z_{f}$. Then, using the definition (36), one can formulate inverse formulas for the electromagnetic-field vectors

(46) $$E=\sqrt{\frac{Z_{f}}{2}}\left(F+F^{*}\right)$$

(47) $$H=\frac{j}{\sqrt{2Z_{f}}}\left(F^{*}-F\right).$$

Furthermore, because of the assumptions (19) and (20), one can define the vector G such as:

(48) $$F=c_{\beta }D_{t}^{1-\beta }G.$$

With the use of power-law property (13), (48) can be written as

(49) $$D_{t}^{\beta }F=c_{\beta }D_{t}^{1}G.$$

Then, assuming that the inverse operator exists for the FO differentiation, (48) can equivalently be written as

(50) $$G=c_{\beta }^{-1}I_{t}^{1-\beta }F.$$

For assumptions given in Section 2.3, the relation between F and G is unambiguous. Therefore, one can write Maxwell’s Equations (23)–(26) as follows:

(51) $$\nabla \times F=j\frac{\partial G}{\partial t}+K$$

(52) ∇·G=ρ.

Hence, based on (50), one can formulate inverse formulas for the electric- and magnetic-flux densities D and B. That is

(53) $$D=\frac{1}{\sqrt{2Z_{f}}}\left(G+G^{*}\right)$$

(54) $$B=j\sqrt{\frac{Z_{f}}{2}}\left(G^{*}-G\right).$$

With the use of (53) and (54), one can define the electromagnetic-momentum density based on Minkowski’s approach [50,51], i.e.,

(55) $$g_{M}=D\times B=jG\times G^{*}.$$

## 4. Plane-Wave Propagation

Let us consider the plane-wave propagation in a free space without sources (ρ=0 and K=0). Let us employ the spatial Fourier transformation (3) to the RS vector, i.e., F(k,t)=F(F(r,t)). For the sake of brevity, the same symbol is employed to denote the Fourier transform, but with the argument k. Application of the spatial Fourier transformation (3) to (40) and (41) gives

(56) $$-k\times F\left(k,t\right)=c_{\beta }^{-1}D_{t}^{\beta }F\left(k,t\right)$$

(57) k·F(k,t)=0.

It implies that k⊥F(k,t). Let us introduce the polarisation vectors $e_{\pm }\left(k\right)$ such as

(58) $$k\times e_{\pm }\left(k\right)=\mp jke_{\pm }\left(k\right)$$

where k=|k|. Eigenvectors of the cross-product (58) differ only by the phase factors. Let us denote $e_{+}\left(k\right)=e\left(k\right)$ and notice that $e_{-}\left(k\right)=e\left(-k\right)=e^{*}\left(k\right)$.

In [25], the solution to IO Maxwell’s Equations (56) and (57) is represented in the RS formalism as follows:

(59) $$F\left(k,t\right)=e_{+}\left(k\right)f_{+}\left(k\right)e^{j\omega t}+e_{-}\left(k\right)f_{-}^{*}\left(-k\right)e^{-j\omega t}.$$

Unfortunately, this is not the case for the considered FO generalization, because components of (59) lead to

(60) $$e_{+}\left(k\right)f_{+}\left(k\right)e^{j\omega t}\left(-jk+c_{\beta }^{-1}{\left(j\omega \right)}^{\beta }\right)=0$$

and

(61) $$e_{-}\left(k\right)f_{-}^{*}\left(k\right)e^{-j\omega t}\left(jk+c_{\beta }^{-1}{\left(-j\omega \right)}^{\beta }\right)=0.$$

It implies that $f_{+}\left(k\right)=f_{-}^{*}\left(k\right)=0$, because none of the complex numbers ${\left(j\omega \right)}^{\beta }$ nor ${\left(-j\omega \right)}^{\beta }$ is purely imaginary for β∈(0,1).

Hence, the general solution to (56) and (57) is written in a more general form

(62) $$F\left(k,t\right)=e_{+}\left(k\right)f_{+}\left(k,t\right)+e_{-}\left(k\right)f_{-}^{*}\left(-k,t\right).$$

The complex conjugate and the minus sign in the second term of (62) stem from our requirement of consistency with the classical theory of the RS vector [25].

Let us consider the first component in (62), i.e., $e_{+}\left(k\right)f_{+}\left(k,t\right)$. It satisfies (56); hence, using the relation $k\times e_{+}\left(k\right)=-jke_{+}\left(k\right)$, one obtains

(63) $$D_{t}^{\beta }f_{+}\left(k,t\right)-jc_{\beta }kf_{+}\left(k,t\right)=0.$$

Similarly, for the component corresponding to the vector $e_{-}\left(k\right)$, one obtains

(64) $$D_{t}^{\beta }f_{-}^{*}\left(-k,t\right)+jc_{\beta }kf_{-}^{*}\left(-k,t\right)=0.$$

By taking the complex conjugate, the last equation can be written as

(65) $$D_{t}^{\beta }f_{-}\left(-k,t\right)-jc_{\beta }kf_{-}\left(-k,t\right)=0.$$

One should note that for both polarization vectors $e_{+}\left(k\right)$, $e_{-}\left(k\right)$ and the fixed vector k, a FO differential equation is obtained

(66) $$D_{t}^{\beta }y\left(t\right)-\lambda y\left(t\right)=0$$

where $\lambda =\lambda \left(k\right)=jc_{\beta }k$. Among the solutions to (66), one may find non-zero functions that are zero on the interval (−∞,a) and which satisfy the Equation (63) on the interval [a,+∞), when $D_{t}^{\beta }$ denotes the Riemann-Lioville derivative of the order β with a base point a∈R. By Theorem 4.1 in [35] (see also (7.2.10) in [52]), the solutions to (66) for λ∈R and t∈[a,+∞), are given by

(67) $$y\left(t\right)=A{\left(t-a\right)}^{\beta -1}E_{\beta ,\beta }\left(\lambda {\left(t-a\right)}^{\beta }\right)$$

where A∈C is a constant. In (67), $E_{\beta ,\beta }$ denotes the Mittag-Leffler function of the second type, i.e., it is the entire complex function given by (see, e.g., (4.1.1) in [52])

(68) $$E_{\beta ,\beta }\left(z\right)=\sum_{n=0}^{\infty }\frac{z^{n}}{\Gamma (\beta n+\beta )}.$$

One should note that in [35,52], the problem with a real value of λ is concerned, but the method of the proof works in the same way also for a complex value of λ. Looking for a causal solution, which is bounded in any set [δ,+∞)⊂R, one has to take the solution for a=0, i.e.,

(69) $$f_{+}\left(k,t\right)={\tilde{f}}_{+}\left(k\right)t^{\beta -1}E_{\beta ,\beta }\left(jc_{\beta }kt^{\beta }\right).$$

To keep the notation shorter, when writing $E_{\beta ,\beta }\left(jc_{\beta }kt^{\beta }\right)$, it is assumed to be equal to 0 for t≤0.

Analogously, the component $e_{-}\left(k\right)f_{-}^{*}\left(-k,t\right)$ with

(70) $$f_{-}\left(-k,t\right)={\tilde{f}}_{-}\left(-k\right)t^{\beta -1}E_{\beta ,\beta }\left(jc_{\beta }kt^{\beta }\right)$$

also satisfies (56).

Therefore, the general solution to (40) for K=0 is given by

(71) $$F\left(r,t\right)=\frac{1}{{\left(2\pi \right)}^{3}}\int_{R^{3}}(e^{-jk\cdot r}e_{+}\left(k\right){\tilde{f}}_{+}\left(k\right)t^{\beta -1}E_{\beta ,\beta }\left(jc_{\beta }kt^{\beta }\right)$$

$$+e^{-jk\cdot r}e_{-}\left(k\right){\tilde{f}}_{-}^{*}\left(-k\right)t^{\beta -1}E_{\beta ,\beta }^{*}\left(jc_{\beta }kt^{\beta }\right))\;d^{3}k.$$

Taking into consideration that $\int_{R^{3}}g\left(-x\right)\;d^{3}x=\int_{R^{3}}g\left(x\right)\;d^{3}x$, $e_{+}\left(k\right)=e\left(k\right)$ and $e_{-}\left(k\right)=e\left(-k\right)$, one can write (71) as follows:

(72) $$F\left(r,t\right)=\frac{1}{{\left(2\pi \right)}^{3}}\int_{R^{3}}e\left(k\right)t^{\beta -1}(e^{-jk\cdot r}{\tilde{f}}_{+}\left(k\right)E_{\beta ,\beta }\left(jc_{\beta }kt^{\beta }\right)$$

$$+e^{jk\cdot r}{\tilde{f}}_{-}^{*}\left(k\right)E_{\beta ,\beta }^{*}\left(-jc_{\beta }kt^{\beta }\right))\;d^{3}k.$$

Let us consider the propagation of attenuated waves along the *z*-direction, i.e., $k\;=\;k\;i_{z}$ and

(73) $$e\left(k\right)=\frac{1}{\sqrt{2}}\left[\begin{array}{c} 1\\ j\\ 0 \end{array}\right].$$

Then, for fixed functions $f_{+}\left(k\right)$ and $f_{-}\left(k\right)$, one can write (71) as follows (remembering that now the spatial Fourier transformation is applied in one dimension only):

(74) $$F\left(z,t\right)=\frac{1}{4\pi }\int_{R}t^{\beta -1}e^{-jkz}\left(f_{+}\left(k\right)E_{\beta ,\beta }\left(jc_{\beta }kt^{\beta }\right)\left[\begin{array}{c} 1\\ j\\ 0 \end{array}\right]+f_{-}^{*}\left(-k\right)E_{\beta ,\beta }^{*}\left(jc_{\beta }kt^{\beta }\right)\left[\begin{array}{c} 1\\ -j\\ 0 \end{array}\right]\right)\;dk.$$

Taking the special case of $f_{+}\left(k\right)=\frac{1}{\sqrt{2}}$ and $f_{-}\left(k\right)=f_{-}^{*}\left(k\right)=-\frac{1}{\sqrt{2}}$, one obtains from (74) for the *x* coordinate

(75) $${\left(F\left(z,t\right)\right)}_{x}=\frac{1}{2\pi }\int_{R}t^{\beta -1}(\cos\left(kz\right)jℑ\left(E_{\beta ,\beta }\left(jc_{\beta }kt^{\beta }\right)\right)+\sin\left(kz\right)ℑ\left(E_{\beta ,\beta }\left(jc_{\beta }kt^{\beta }\right)\right)dk.$$

Let us now consider real and imaginary parts of $t^{\beta -1}E_{\beta ,\beta }\left(jc_{\beta }kt^{\beta }\right)$. As one can see, these may be represented as

(76) $$t^{\beta -1}ℜ(E_{\beta ,\beta }\left(jc_{\beta }kt^{\beta }\right)=t^{\beta -1}E_{2\beta ,\beta }\left(-c_{\beta }^{2}k^{2}t^{2\beta }\right)$$

(77) $$t^{\beta -1}ℑ(E_{\beta ,\beta }\left(jc_{\beta }kt^{\beta }\right)=t^{2\beta -1}c_{\beta }kE_{2\beta ,2\beta }\left(-c_{\beta }^{2}k^{2}t^{2\beta }\right).$$

Hence, the real part of ${\left(F\left(z,t\right)\right)}_{x}$, i.e., the electric-field component, can be written as

(78) $$E_{x}\left(z,t\right)=\frac{1}{2\pi }t^{2\beta -1}c_{\beta }\int_{R}kE_{2\beta ,2\beta }\left(-c_{\beta }^{2}k^{2}t^{2\beta }\right)\sin\left(kz\right)dk.$$

This is Green’s function, which is given in [53] for, so called, signalling problem for the diffusion-wave equation

(79) $$D_{t}^{2\beta }u\left(x,t\right)-c_{\beta }^{2}D_{x}^{2}u\left(x,t\right)=0$$

with an appropriate boundary condition (cf. formula (17) in [53], where the normalized problem with $c_{\beta }=1$ is concerned). In [53], Green’s function is given by

(80) $$G_{s}\left(x,t\right)=\frac{2t^{2\beta -1}}{\pi }\int_{0}^{\infty }kE_{2\beta ,2\beta }\left(-k^{2}c_{\beta }^{2}t^{2\beta }\right)\sin\left(kx\right)dk$$

and differs from (78) by a multiplicative constant only.

The formula (80) is one of many equivalent representations including the Formula (72) from [54], which is taken from [45,47], and used in our numerical simulations. These two formulations are actually closely related through the integration by parts formula.

Let us also consider the solution (71), which is the boundary case for β=1 and $c_{\beta }=c$. Let us take $f_{+}\left(k\right)=f_{-}\left(-k\right)=f_{-}^{*}\left(-k\right)=\frac{1}{\sqrt{2}}$. Hence, one obtains classical unattenuated solutions in the form:

(81) $$F\left(z,t\right)=\frac{1}{4\pi }\int_{R}e^{-jkz}e^{jckt}\left[\begin{array}{c} 1\\ j\\ 0 \end{array}\right]+e^{-jkz}e^{-jckt}\left[\begin{array}{c} 1\\ -j\\ 0 \end{array}\right]\;dk=$$

$$\frac{1}{2}\left(\delta \left(z-ct\right)\left[\begin{array}{c} 1\\ j\\ 0 \end{array}\right]+\delta \left(z+ct\right)\left[\begin{array}{c} 1\\ -j\\ 0 \end{array}\right]\right).$$

Consistency with solutions for IO Maxwell’s equations confirms correctness of our derivations.

**Figure 1:**
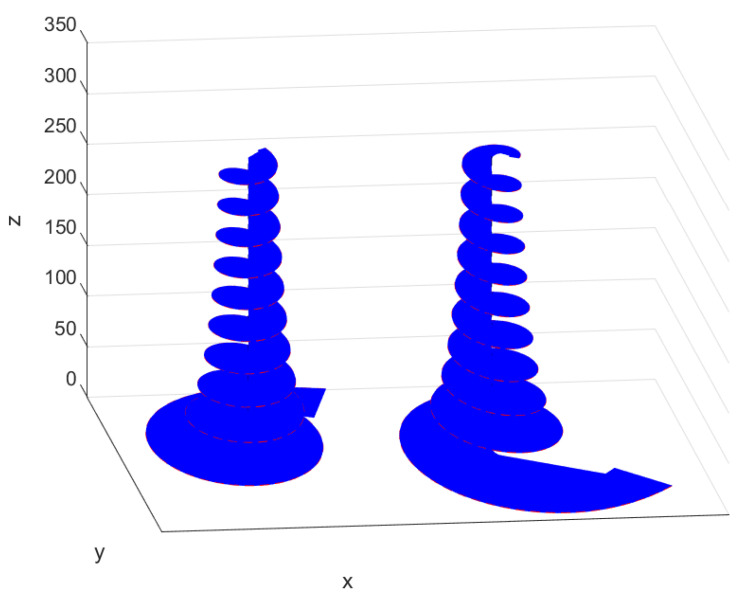
Rotating polarisation vectors ($k=ki_{z}$) related to $f_{+}$ (on **left**) and $f_{-}$ (on **right**) components for β=0.6.

Now, one can identify the left- and right-handed circularly polarized attenuated waves propagating in the *z*-direction, which are related with $f_{+}$ and $f_{-}$ functions within the general solution (72), respectively. In Figure 1, the rotating polarisation vectors $e\left(k\right)t^{\beta -1}e^{-jk\cdot r}E_{\beta ,\beta }\left(jc_{\beta }kt^{\beta }\right)$ and $e\left(k\right)t^{\beta -1}e^{jk\cdot r}E_{\beta ,\beta }^{*}\left(-jc_{\beta }kt^{\beta }\right)$ (where $k=ki_{z}$) are presented for these components, which create helical surfaces. As seen, for both components related to $f_{+}$ and $f_{-}$ functions, the wave amplitude is attenuated and helices rotate in opposite directions.

## 5. Fundamental Solutions

Maxwell’s Equations (40) and (41) lead to the following equation

(82) $$\nabla \left(\nabla \cdot F\right)-\nabla^{2}F=-c_{\beta }^{-2}D_{t}^{2\beta }F+jc_{\beta }^{-1}D_{t}^{\beta }K+\nabla \times K$$

and eventually

(83) $$\nabla^{2}F-c_{\beta }^{-2}D_{t}^{2\beta }F=c_{\beta }D_{t}^{1-\beta }\nabla \rho -jc_{\beta }^{-1}D_{t}^{\beta }K-\nabla \times K.$$

Let us consider the TF diffusion-wave equation in free space without sources

(84) $$\nabla^{2}F-c_{\beta }^{-2}D_{t}^{2\beta }F=0.$$

Let us solve (84) by separating the variables, i.e., assuming that

(85) $$F=(F_{x},F_{y},F_{z})$$

and

(86) $$F_{i}=\Psi_{i}\left(r\right)T_{i}\left(t\right)$$

where i∈{x,y,z} for the Cartesian coordinate system. Each of the components $\Psi_{i}\left(r\right)T_{i}\left(t\right)$ satisfies the scalar equation as follows:

(87) $$c_{\beta }^{2}\frac{\nabla^{2}\Psi_{i}\left(r\right)}{\Psi_{i}\left(r\right)}=\frac{D_{t}^{2\beta }T_{i}\left(t\right)}{T_{i}\left(t\right)}.$$

Naturally, it is assumed that $\Psi_{i}\left(r\right)\neq 0$ and $T_{i}\left(t\right)\neq 0$ in (87). Since both sides of (87) are functions of different variables, then both sides of (87) are equal to the constant $q_{i}^{2}$. Hence, (84) is equivalent to the following set of equations

(88) $$\nabla^{2}\Psi_{i}-\frac{q_{i}^{2}}{c_{\beta }^{2}}\Psi_{i}=0$$

(89) $$D_{t}^{2\beta }T_{i}\left(t\right)-q_{i}^{2}T_{i}\left(t\right)=0.$$

Equation (89) is satisfied by the functions $e^{j\omega_{i}t}$ when $q_{i}^{2}={\left(j\omega_{i}\right)}^{2\beta }$ and $e^{-j\omega_{i}t}$ when $q_{i}^{2}={\left(-j\omega_{i}\right)}^{2\beta }$; hence, the general solution to the *i*-th coordinate of (84) can be written as

(90) $$F_{i}={\tilde{F}}_{i,-}e^{j\omega_{i}t}+{\tilde{F}}_{i,+}e^{-j\omega_{i}t}.$$

To obtain the solution to the vector Equation (84) from (90), the same frequency (i.e., $\omega =\omega_{x}=\omega_{y}=\omega_{z}$) for all coordinate components has to be assumed. Then, one obtains

(91) $$F={\tilde{F}}_{-}e^{j\omega t}+{\tilde{F}}_{+}e^{-j\omega t}$$

where ${\tilde{F}}_{-}={\tilde{F}}_{-}\left(r\right)$ and ${\tilde{F}}_{+}={\tilde{F}}_{+}\left(r\right)$. Assuming that ω∈R, the solution (91) can be reduced to the phasor representation of the RS vector, i.e., $F=\tilde{F}e^{j\omega t}$ where $\tilde{F}=\tilde{F}\left(r\right)$ is a complex vector. Then, one obtains the following stationary equation, which depends on the angular frequency ω only:

(92) $$\nabla^{2}\tilde{F}-c_{\beta }^{-2}{\left(j\omega \right)}^{2\beta }\tilde{F}=0.$$

In the case of plane wave propagating in $R^{3}$, one can assume that the propagation takes place along the *z*-direction. It corresponds to the vector field $\tilde{F}$ given by

(93) $$\tilde{F}\left(r\right)=\tilde{F}\left(z\right)e_{z}$$

where $\tilde{F}\left(z\right)$ is the scalar complex-valued function and $e_{z}$ is the corresponding unit vector given by (73). Then, inserting (93) into (92), one obtains the corresponding 1-D equation for an unknown complex-valued function $\tilde{F}\left(z\right)$

(94) $$\frac{d^{2}\tilde{F}\left(z\right)}{dz^{2}}-c_{\beta }^{-2}{\left(j\omega \right)}^{2\beta }\tilde{F}\left(z\right)=0$$

whose the solution is given by

(95) $$\tilde{F}\left(z\right)={\tilde{F}}_{+}e^{-z\xi }+{\tilde{F}}_{-}e^{z\xi }$$

where $\xi =c_{\beta }^{-1}{\left(j\omega \right)}^{\beta }$ and $F_{+}$ and $F_{-}$ are certain complex constants. Hence, for a fixed *z*, one obtains the scalar transfer function in the frequency domain

(96) $$G_{z}\left(\omega \right)={\tilde{F}}_{+}e^{-zc_{\beta }^{-1}{\left(j\omega \right)}^{\beta }}$$

where ${\tilde{F}}_{+}\in C$ is a certain complex constant. Its value does not influence the causality of the transform, i.e., it can be demonstrated that the transform $e^{-zc_{\beta }^{-1}{\left(j\omega \right)}^{\beta }}$ is causal iff β∈(0,1) [55]. However, this transform is not relativistically causal, i.e., when t<0 then the inverse Fourier transform of $e^{-zc_{\beta }^{-1}{\left(j\omega \right)}^{\beta }}$ is equal to zero, whilst it is different then zero for $e^{-zc_{\beta }^{-1}{\left(j\omega \right)}^{\beta }}e^{j\omega zc^{-1}}$.

One may also consider the radially symmetric solutions to (92). Hence, let us assume that the solution $\tilde{F}$ is given by

(97) $$\tilde{F}\left(x,y,z\right)=f\left(\sqrt{x^{2}+y^{2}+z^{2}}\right)e_{R}=f\left(R\right)e_{R}$$

where $e_{R}$ is the corresponding unit vector in spherical system. Then, one obtains

(98) $$\Delta \tilde{F}\left(x,y,z\right)=f^{\prime \prime }\left(R\right)+\frac{2}{R}f^{\prime }\left(R\right)=\frac{1}{R}\frac{d}{dR}\left(Rf^{\prime }\left(R\right)+f\left(R\right)\right)=\frac{1}{R}\frac{d^{2}}{dR^{2}}\left(Rf(R)\right)$$

where $R=\sqrt{x^{2}+y^{2}+z^{2}}$. Then, the 3-D Equation (92) takes the form

(99) $$\frac{d^{2}}{dR^{2}}\left(Rf(R)\right)-c_{\beta }^{-2}{\left(j\omega \right)}^{2\beta }\left(Rf\left(R\right)\right)=0$$

and the solution Rf(R) is given by

(100) $$Rf\left(R\right)={\tilde{F}}_{+}e^{-Rc_{\beta }^{-1}{\left(j\omega \right)}^{\beta }}.$$

It gives

(101) $$f\left(R\right)=\frac{1}{R}{\tilde{F}}_{+}e^{-Rc_{\beta }^{-1}{\left(j\omega \right)}^{\beta }}.$$

For a fixed R>0, one can write the transfer function as

(102) $$G_{R}\left(\omega \right)=\frac{1}{4\pi }\frac{1}{R}{\tilde{F}}_{+}e^{-Rc_{\beta }^{-1}{\left(j\omega \right)}^{\beta }}$$

where the normalizing factor appears following Section 2.3.1 of [56] and the dependence on ω has the same form as before. Hence, it is a causal transform iff β∈(0,1).

One may also consider the solution $\tilde{F}$ with cylindrical symmetry, i.e., given by

(103) $$\tilde{F}\left(x,y,z\right)=f\left(\sqrt{x^{2}+y^{2}},z\right)e_{r}=f\left(r,z\right)e_{r},$$

where $e_{r}$ is the correspoding unit vector in cylindrical system. With the assumption that the wave propagates in the xy plane (i.e., with z=const), one obtains

(104) $$\Delta \tilde{F}\left(x,y,z\right)=f^{\prime \prime }\left(r\right)+\frac{1}{r}f^{\prime }\left(r\right)$$

where $r=\sqrt{x^{2}+y^{2}}$. Then, the 3-D Equation (92) takes the form

(105) $$f^{\prime \prime }\left(r\right)+\frac{1}{r}f^{\prime }\left(r\right)-c_{\beta }^{-2}{\left(j\omega \right)}^{2\beta }f\left(r\right)=0$$

which can be written as

(106) $$r^{2}f^{\prime \prime }\left(r\right)+rf^{\prime }\left(r\right)-c_{\beta }^{-2}{\left(j\omega \right)}^{2\beta }r^{2}f\left(r\right)=0.$$

Equation (106) is known as the Bessel equation of the order α=0. Its solutions are known as cylinder or Bessel functions (see Section 3.9 in [57] for a recursive definition of cylinder functions and Section 4.3, Equations (3) and (4), for solutions of the Bessel Equation (105) as well as Sections 8.4 and 8.5 in [58]). As one can notice, (105) corresponds to the Equation (3) in Section 4.3 in Watson’s book [57] with $c={\left(j\omega \right)}^{\beta }/c_{\beta }$ and p=−1/2. Hence, the solutions to (105) are functions given by

(107) $$f\left(r\right)=Z_{0}\left(j{\left(j\omega \right)}^{\beta }r/c_{\beta }\right)$$

where $Z_{0}$ is any Bessel function of the order ν=0 (as in Sections 8.4 and 8.5 of [58], or the cylinder function $C_{0}$ from Watson’s book [57]). As a special case of cylinder functions, one can consider (see 8.401 in [58]) the Bessel functions of the first kind $J_{0}\left(z\right)$, the Bessel functions of the second kind $Y_{0}\left(z\right)$ and the Hankel functions $H_{0}^{\left(1\right)}\left(z\right)$, $H_{0}^{\left(2\right)}\left(z\right)$ (also called the Bessel functions of the third kind). Each of the pairs ($J_{0}\left(j{\left(j\omega \right)}^{\beta }r/c_{\beta }\right)$, $Y_{0}\left(j{\left(j\omega \right)}^{\beta }r/c_{\beta }\right)$) or ($H_{0}^{\left(1\right)}\left(j{\left(j\omega \right)}^{\beta }r/c_{\beta }\right)$, $H_{0}^{\left(2\right)}\left(j{\left(j\omega \right)}^{\beta }r/c_{\beta }\right)$) forms a fundamental system of solutions to (105). In the considered case, we take a function that tends to 0 as r→+∞. We assume that $f\left(r\right)=J_{0}\left(j{\left(j\omega \right)}^{\beta }r/c_{\beta }\right)$, where Bessel function $J_{0}$ is given by the series

(108) $$J_{0}\left(x\right)=\sum_{k=0}^{+\infty }{\left(-1\right)}^{k}\frac{1}{\Gamma {\left(k+1\right)}^{2}}{\left(\frac{x}{2}\right)}^{2k}.$$

Hence, for a fixed r>0, the transfer function is given by

(109) $$G_{r}\left(\omega \right)=-\frac{j}{4}{\tilde{F}}_{+}J_{0}\left(j{\left(j\omega \right)}^{\beta }r/c_{\beta }\right).$$

The Bessel function is multiplied by a factor $-\frac{j}{4}$, which comes from the normalization as in Section 2.3.2 in [56] (Hankel functions are used in [56], but one should remember that $J_{0}\left(z\right)=\frac{1}{2}\left(H_{0}^{\left(1\right)}\left(z\right)+H_{0}^{\left(2\right)}\left(z\right)\right)$ which makes the condition valid for $J_{0}$ as well).

**Figure 2:**
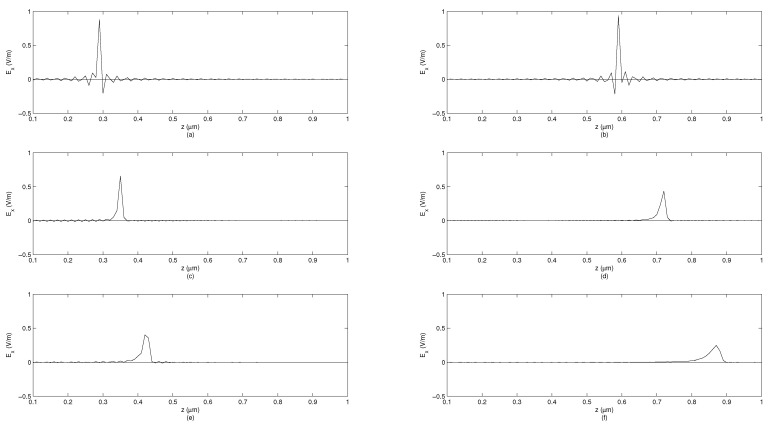
System response depending on distance from source for plane-wave propagation along *z* direction in FOM, signal measured at time points $t_{1}=1fs$ and $t_{2}=2fs$. (**a**) β=1, $t=t_{1}$. (**b**) β=1, $t=t_{2}$. (**c**) β=0.995, $t=t_{1}$. (**d**) β=0.995, $t=t_{2}$. (**e**) β=0.99, $t=t_{1}$. (**f**) β=0.99, $t=t_{2}$.

**Figure 3:**
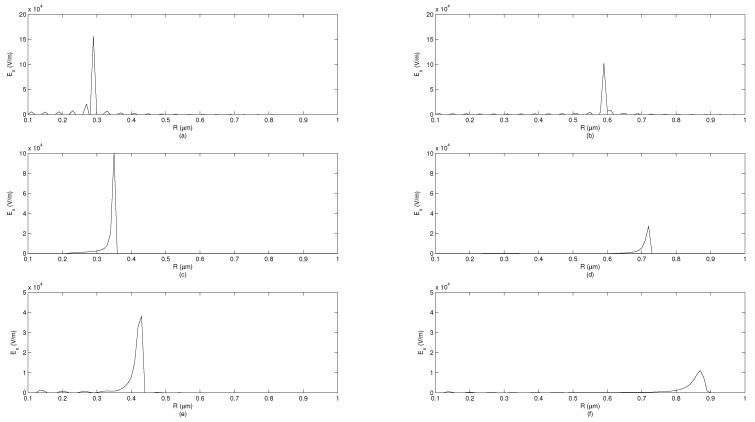
System response depending on distance from source for radial symmetry in FOM, signal measured at time points $t_{1}=1fs$ and $t_{2}=2fs$. (**a**) β=1, $t=t_{1}$. (**b**) β=1, $t=t_{2}$. (**c**) β=0.995, $t=t_{1}$. (**d**) β=0.995, $t=t_{2}$. (**e**) β=0.99, $t=t_{1}$. (**f**) β=0.99, $t=t_{2}$.

**Figure 4:**
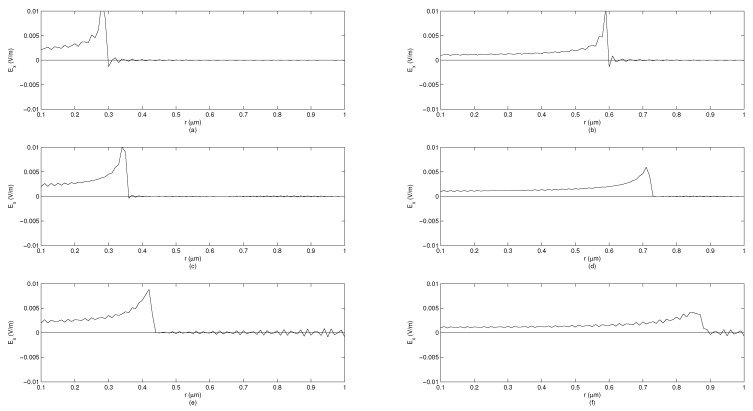
System response depending on distance from source for cylindrical symmetry in FOM, signal measured at time points $t_{1}=1fs$ and $t_{2}=2fs$. (**a**) β=1, $t=t_{1}$. (**b**) β=1, $t=t_{2}$. (**c**) β=0.995, $t=t_{1}$. (**d**) β=0.995, $t=t_{2}$. (**e**) β=0.99, $t=t_{1}$. (**f**) β=0.99, $t=t_{2}$.

To illustrate differences between solutions for plane wave, radial, and cylindrical symmetries, we performed simulations showing the electric field observed at certain moments in time at spatial points of varying distance from the source. Three transfer functions (96), (102) and (109) are considered. The algorithm of computations is described in Section 5 of [34] and verified against accurate solutions [59]. The simulation shows the system response to the unit-impulse excitation (which approximates Dirac’s delta distribution) in two time moments $t_{1}=1fs$ and $t_{2}=2fs$ at the distance varying from 0.1–1 μm. The simulations are performed assuming the sampling period $T_{s}=\frac{1}{50\cdot 750}10^{-12}s$. It is assumed that the value of $c_{\beta }$ is equal to *c*, but expressed in appropriate units, i.e., $\frac{m}{s^{\beta }}$. The simulations are performed for β∈{1,0.995,0.99}. Figure 2 corresponds to the plane-wave propagation, Figure 3 corresponds to the radial-symmetry case, while Figure 4 corresponds to the cylindrical symmetry case. In each figure, for β<1, an inclined wavefront is observed with the maximum of a pulse propagating with finite velocity. For the radial-symmetry case, the values of the plotted function are much larger due to singularity in the formula (102) for R=0. As one can notice, the electric-field intensity is different than zero before the arrival of the wavefront maximum. This feature means that fundamental solutions significantly differ from Green’s functions in IO electrodynamics. The disturbance spreads infinitely fast, which makes the solutions similar to the ones known for the TF diffusion-wave equation being non-relativistic, similarly to the standard diffusion equation. As one can see in all the cases, the decreasing derivative order (i.e., when systems become ‘more fractional’), the amplitude of the system response decreases but the pulse width is increasing. Furthermore, the ‘tails’ of pulses are increasing, which represent the memory effects of FOMs.

## 6. Power Conservation

Let us consider the energy-balance equation (i.e., Poynting’s theorem) in TF electrodynamics formulated in the time domain (without electric and magnetic currents and Joule’s heating, i.e., J=0 and M=0, for the sake of brevity) [60]

(110) $$\nabla \cdot \left(E\times H\right)+\epsilon_{\beta }E\cdot D_{t}^{\beta }E+\mu_{\beta }H\cdot D_{t}^{\beta }H=0.$$

Then, using (46) and (47), one obtains this theorem formulated based on the RS vector

(111) $$j\nabla \cdot \left(F\times F^{*}\right)+c_{\beta }^{-1}\left(F\cdot D_{t}^{\beta }F^{*}+F^{*}\cdot D_{t}^{\beta }F\right)=0.$$

Equation (111) can be rewritten as

(112) $$\nabla \cdot S+\frac{\partial u}{\partial t}=0$$

where

(113) $$\frac{\partial u}{\partial t}=c_{\beta }^{-1}\left(F\cdot D_{t}^{\beta }F^{*}+F^{*}\cdot D_{t}^{\beta }F\right)$$

is the density of accumulated and dissipated power of the field and the medium described by FOM. Hence,

(114) $$u=c_{\beta }^{-1}I_{t}^{1}\left(F\cdot D_{t}^{\beta }F^{*}+F^{*}\cdot D_{t}^{\beta }F\right)$$

denotes the density of electromagnetic-field energy, which is spent to create the field and change the momentum of medium. This integral exists because F is continuous and bounded as well as $D_{t}^{\beta }F$ belongs to $L^{1}\left(R\right)$. It depends upon the entire history of the process, which is typical for the TF electrodynamics of media with memory but unnecessary for a vacuum [61]. Important is to notice that we cannot distinguish in (113) and (114) power and energy components, respectively, which are conserved in the field and dissipated in the medium described by FOM.

For a vacuum when β=1, (114) gives the standard formula for the energy density in classical IO electrodynamics

(115) $$u=c^{-1}F\cdot F^{*}=cG\cdot G^{*}=\frac{1}{2}(\epsilon_{0}{\left|E\right|}^{2}+\mu_{0}{\left|H\right|}^{2}).$$

As one can notice, the energy density formula (115) is local in time for β=1. The case of β∈(0,1) is totally different. The formula (114) is nonlocal in time not only because of the integral $I_{t}^{1}$, but also due to nonlocality of fractional derivatives $D_{t}^{\beta }$. Let us demonstrate that nonlocal nature of the derivative $D_{t}^{\beta }$ introduces appropriate memory effects into the energy-density formula (114). Consider now a local in time RS vector as an ‘input signal’ to (114), which approximates Dirac’s delta distribution

(116) $$F_{T}=\frac{1}{T}\left\{\begin{array}{cc} v & t\in [0,T]\\ 0 & t\notin [0,T] \end{array}\right.$$

where v is a fixed vector such as $v\cdot v^{*}={\left|v\right|}^{2}=1$ and *T* denotes an approximation parameter. One should note that although $F_{T}$ is not a solution to the diffusion-wave Equation (84), it allows to analyse (114) in terms of memory effects. Let us show that $F_{T}$ influences the right-hand side of (114) not only for t∈[0,T] but for all times t>0, when β∈(0,1). It means that memory effects can be observed in this case for t>T. One can see that

(117) $$D_{t}^{\beta }F_{T}=\frac{1}{T\Gamma (1-\beta )}t^{-\beta }v$$

and

(118) $$D_{t}^{\beta }F_{T}^{*}=\frac{1}{T\Gamma (1-\beta )}t^{-\beta }v^{*}$$

for t∈[0,T]. Hence, for t≥T,

(119) $$\int_{-\infty }^{t}\left(F_{T}\cdot D_{\tau }^{\beta }F_{T}^{*}+F_{T}^{*}\cdot D_{\tau }^{\beta }F_{T}\right)d\tau =\int_{0}^{T}\frac{2\tau^{-\beta }}{T^{2}\Gamma \left(1-\beta \right)}d\tau =\frac{2}{\left(1-\beta \right)\Gamma \left(1-\beta \right)T^{1+\beta }}.$$

**Figure 5:**
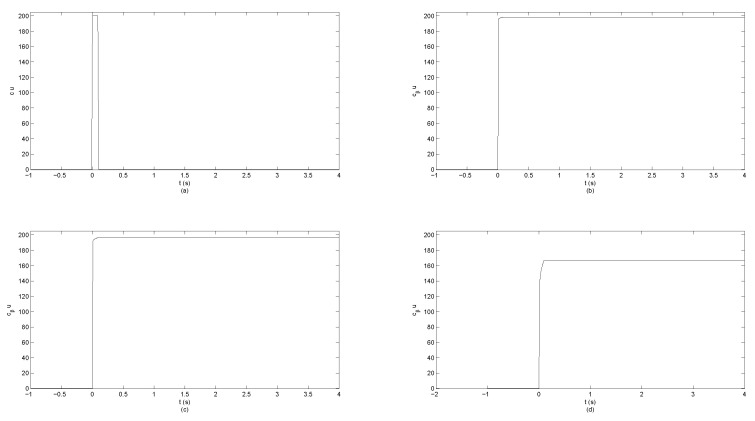
Memory effect in energy density *u* (multiplied by $c_{\beta }$). (**a**) Local in time effect corresponding to β=1. (**b**) β=0.995. (**c**) β=0.95. (**d**) β=0.9.

It shows that the effect of ‘input signal’ $F_{T}$ with compact support is accumulated in the density of electromagnetic-field energy up to t→+∞. Memory effects, for T=0.1 s, are presented in Figure 5.

As one can see, for β=1, the energy density *u* is nonzero only for t∈[0,T], when the RS vector $F_{T}$ is nonzero as well. However, for β∈(0,1), the energy density *u* saturates when nonzero $F_{T}$ appears. The amplitude of saturation decreases when β is decreased. One should note that for β=1 and the discontinuous function $F_{T}$, we may not use the formula (115), because we may not apply the Leibniz product rule to the non-differentiable functions. Hence, we need to directly apply the formula (114) in a distributional sense.

Suppose now that $V\subset R^{3}$ is a compact volume with the boundary *S* being the piecewise smooth surface. Hence, with the use of Gauss’s theorem and (44), the theorem (111) can be written in the integral form as

(120) $$\oint_{S=\partial V}S\cdot \;da+c_{\beta }^{-1}\int_{V}\left(F\cdot D_{t}^{\beta }F^{*}+F^{*}\cdot D_{t}^{\beta }F\right)\;dv=0.$$

Equation (120) describes the balance of the power dissipated and stored in the electromagnetic field in the considered volume *V*. Since $S\in R^{3}$, then one obtains

(121) $$\oint_{S=\partial V}S\cdot \;da+c_{\beta }^{-1}ℜ\int_{V}\left(F\cdot D_{t}^{\beta }F^{*}+F^{*}\cdot D_{t}^{\beta }F\right)\;dv=0$$

(122) $$ℑ\int_{V}\left(F\cdot D_{t}^{\beta }F^{*}+F^{*}\cdot D_{t}^{\beta }F\right)\;dv=0.$$

Equation (122) is always valid for $S\in R^{3}$, because

(123) $$D_{t}^{\beta }F=\frac{1}{\sqrt{2}}\left(\frac{D_{t}^{\beta }E}{\sqrt{Z_{f}}}+j\left(D_{t}^{\beta }H\right)\sqrt{Z_{f}}\right).$$

Hence, the imaginary part of expression under the integral (122) is identically equal to zero. In (121), the volume integral involves dot products between E/H fields and their FO derivatives. On the other hand, one can notice in (122) that the volume integral involves dot products between E/H fields and FO derivatives of H/E fields, respectively. It can be treated, to some extent, as the orthogonality condition between the components of the RS vector and their FO derivatives.

With the use of (114), one can calculate the total electromagnetic-field energy (i.e., Hamiltonian of the field and the medium) as follows:

(124) $$H=\int_{V}u\;dv.$$

## 7. Momentum Conservation

Let us formulate the momentum-conservation theorem [48,49] in TF electrodynamics. Let us assume that M=0 and $\rho_{m}=0$ in (23)–(26). Then, one can formulate the momentum-balance equation without using the definition of Lorentz force and the constitutive relations [62,63]:

(125) $$\nabla \cdot \left(DE-\frac{1}{2}\left(D\cdot E\right)I+BH-\frac{1}{2}\left(B\cdot H\right)I\right)-\frac{\partial }{\partial t}\left(D\times B\right)=$$

$$\rho_{e}E+J\times B-\frac{1}{2}\left((\nabla D)\cdot E-(\nabla E)\cdot D+(\nabla B)\cdot H-(\nabla H)\cdot B\right).$$

Lack of symbol between vectors denotes a tensor or dyadic product. The right-hand side of (125) represents the force density which includes, besides the Lorentz force with total fields, other force densities, e.g., Helmholtz’s for gases [62]. Above equation can be written using the RS vector as follows:

(126) $$\nabla \cdot T-j\frac{\partial }{\partial t}G\times G^{*}=f.$$

In (126), T denotes the stress tensor, i.e.,

(127) $$T=DE-\frac{1}{2}\left(D\cdot E\right)I+BH-\frac{1}{2}\left(B\cdot H\right)I$$

and f denotes the force density, i.e.,

(128) $$f=\rho_{e}E+J\times B$$

$$-\frac{1}{2}\left(\epsilon_{\beta }\left[\left(\nabla I_{t}^{1-\beta }E\right)\cdot E-\left(\nabla E\right)\cdot I_{t}^{1-\beta }E\right]+\mu_{\beta }\left[\left(\nabla I_{t}^{1-\beta }H\right)\cdot H-\left(\nabla H\right)\cdot I_{t}^{1-\beta }H\right]\right).$$

For β=1, (128) reduces to the standard formula for the Lorentz force, i.e.,

(129) $$f=\rho_{e}E+J\times B.$$

For β≠1, if $\left(\nabla I_{t}^{1-\beta }E\right)\cdot E\neq \left(\nabla E\right)\cdot I_{t}^{1-\beta }E$ or $\left(\nabla I_{t}^{1-\beta }H\right)\cdot H\neq \left(\nabla H\right)\cdot I_{t}^{1-\beta }H$, then additional contribution to the force density is obtained due to the application of FOM.

Suppose now that $V\subset R^{3}$ is a compact volume with the boundary *S* being the piecewise smooth surface. Then, using Gauss’s theorem, the formula (126) can be written in the integral form as

(130) $$\int_{V}\left(f+j\frac{\partial }{\partial t}\left(G\times G^{*}\right)\right)\;dv=\oint_{S=\partial V}T\cdot \;da.$$

Equation (130) describes the momentum conservation in the electromagnetic field in the considered volume *V*, i.e., the total change of the mechanical (i.e., related with an electromagnetic medium) and electromagnetic-field momentum is equal to the momentum flowing into and out of the volume *V*.

Finally, one can calculate the total electromagnetic-field momentum $G_{M}$ and the total angular momentum $L_{M}$ as follows:

(131) $$G_{M}=\int_{V}g_{M}\;dv$$

(132) $$L_{M}=\int_{V}r\times g_{M}\;dv.$$

Although the stress tensor (127) is asymmetric, one can formulate the balance equation of angular momentum using the approach proposed in [64].

## 8. Uniqueness of Solutions

Let us consider the boundary *S* of the volume $V\subset R^{3}$, which is the piecewise smooth surface. Then, let us define either the tangential electric- or magnetic-field intensity. Now, suppose that two solutions $F_{1}$ and $F_{2}$ of TF Maxwell’s equations exist in *V*. Hence, the solution $F_{d}=F_{1}-F_{2}$ of TF Maxwell’s Equations (40) and (41) is obtained assuming that either the tangential electric or magnetic field is equal to zero at the boundary *S*. The function $F_{d}$ satisfies homogeneous Equations (40) and (41)

(133) $$\nabla \times F_{d}=jc_{\beta }^{-1}D_{t}^{\beta }F_{d}$$

(134) $$\nabla \cdot F_{d}=0.$$

By applying the Fourier transformation to both sides of (133), one obtains

(135) $$\nabla \times {\tilde{F}}_{d}=jc_{\beta }^{-1}{\left(j\omega \right)}^{\beta }{\tilde{F}}_{d}$$

where ${\tilde{F}}_{d}\left(r,\omega \right)=F\left(F_{d}\left(r,t\right)\right)$. Taking the complex conjugates, one obtains

(136) $$\nabla \times {\tilde{F}}_{d}^{*}=-jc_{\beta }^{-1}{\left(-j\omega \right)}^{\beta }{\tilde{F}}_{d}^{*}.$$

When the operators $\left({\tilde{F}}_{d}^{*}\cdot \right)$ and $\left({\tilde{F}}_{d}\cdot \right)$ are respectively applied to (135) and (136), and then these equations are subtracted, one obtains

(137) $$\nabla \cdot \left({\tilde{F}}_{d}\times {\tilde{F}}_{d}^{*}\right)=jc_{\beta }^{-1}\left({\left(j\omega \right)}^{\beta }+{\left(-j\omega \right)}^{\beta }\right){\left|{\tilde{F}}_{d}\right|}^{2}.$$

Now, using Gauss’s theorem, one obtains

(138) $$0=jc_{\beta }^{-1}\left({\left(j\omega \right)}^{\beta }+{\left(-j\omega \right)}^{\beta }\right)\int_{V}{\left|{\tilde{F}}_{d}\right|}^{2}dv.$$

Since $\left({\left(j\omega \right)}^{\beta }+{\left(-j\omega \right)}^{\beta }\right)=2{\left|\omega \right|}^{\beta }\cos\left(\beta \frac{\pi }{2}\mathrm{sgn}\left(\omega \right)\right)$, there is

(139) $$0=2jc_{\beta }^{-1}{\left|\omega \right|}^{\beta }\cos\left(\beta \frac{\pi }{2}\mathrm{sgn}\left(\omega \right)\right)\int_{V}{\left|{\tilde{F}}_{d}\right|}^{2}dv.$$

Hence, for β∈(0,1), there is $\int_{V}{\left|{\tilde{F}}_{d}\right|}^{2}dv=0$ and consequently ${\tilde{F}}_{d}=0$. Finally, one obtains that ${\tilde{F}}_{1}={\tilde{F}}_{2}$. As one can expect, it means that also for TF Maxwell’s equations in the RS representation, the solutions are unique for assumed boundary conditions.

## 9. Reciprocity

Reciprocity describes an electromagnetic system, whose a response to a source is unchanged when the source and the measurer are interchanged [65]. We extend this property hereby towards the FO electrodynamics, defined based on the RS vector, by deriving the Lorentz reciprocity theorem [54].

Let us consider a volume *V* of the medium described by FOM, which includes sources K. Then, taking the Fourier transformation with respect to the variable *t*, curl Maxwell’s Equation (40) can be written as

(140) $$\nabla \times \tilde{F}=jc_{\beta }^{-1}{\left(j\omega \right)}^{\beta }\tilde{F}+\tilde{K}$$

where $\tilde{F}\left(r,\omega \right)=F\left(F\left(r,t\right)\right)$ and $\tilde{K}\left(r,\omega \right)=F\left(K\left(r,t\right)\right)$. Let us consider two solutions of FO Maxwell’s equations ${\tilde{F}}_{i}$ existing for current sources ${\tilde{K}}_{i}$ in the volume *V* (i=1,2). Then, one obtains

(141) $$\nabla \times {\tilde{F}}_{1}=jc_{\beta }^{-1}{\left(j\omega \right)}^{\beta }{\tilde{F}}_{1}+{\tilde{K}}_{1}$$

(142) $$\nabla \times {\tilde{F}}_{2}=jc_{\beta }^{-1}{\left(j\omega \right)}^{\beta }{\tilde{F}}_{2}+{\tilde{K}}_{2}.$$

Taking into consideration that

(143) $$\nabla \cdot \left({\tilde{F}}_{1}\times {\tilde{F}}_{2}\right)={\tilde{F}}_{2}\cdot \left(\nabla \times {\tilde{F}}_{1}\right)-{\tilde{F}}_{1}\cdot \left(\nabla \times {\tilde{F}}_{2}\right)$$

one obtains

(144) $$\nabla \cdot \left({\tilde{F}}_{1}\times {\tilde{F}}_{2}\right)={\tilde{F}}_{2}\cdot {\tilde{K}}_{1}-{\tilde{F}}_{1}\cdot {\tilde{K}}_{2}.$$

Equation (144) is a differential form of the Lorentz reciprocity theorem in TF electrodynamics, formulated making use of the RS vector. Applying Gauss’s theorem to (144), one obtains the integral form of the Lorentz reciprocity theorem

(145) $$\oint_{S=\partial V}\left({\tilde{F}}_{1}\times {\tilde{F}}_{2}\right)\cdot \;da=\int_{V}\left({\tilde{F}}_{2}\cdot {\tilde{K}}_{1}-{\tilde{F}}_{1}\cdot {\tilde{K}}_{2}\right)\;dv.$$

Let us assume that the surface integral in (145) vanishes. It is a realistic assumption, because the surface of integration can be extended to infinity from the sources. Then, for β=1, the electric and magnetic fields are related by the plane-wave formulas $Z_{f}{\tilde{H}}_{1/2}\;=\;n\times {\tilde{E}}_{1/2}$ and $n\cdot {\tilde{E}}_{1/2}=0$, where n is the unit vector normal to the integration surface *S* pointing outwards. However, for 0<β<1, the electromagnetic field is exponentially attenuated towards zero, when the surface of integration is extended to infinity from the sources. Hence, one obtains

(146) $$\int_{V}\left({\tilde{F}}_{2}\cdot {\tilde{K}}_{1}\right)\;dv=\int_{V}\left({\tilde{F}}_{1}\cdot {\tilde{K}}_{2}\right)\;dv.$$

This means that volume integrals of the dot products of the electromagnetic fields ${\tilde{F}}_{1/2}$ (resulting from the excitations ${\tilde{K}}_{1/2}$ and measured in their positions) and the current sources ${\tilde{K}}_{2/1}$ are the same.

## 10. Causality

Now let us quickly review the causality. The complex-valued transfer function/distribution in the frequency domain G:R→C is considered, which is the Fourier transform of a certain time-domain function/distribution g:R→C. One should note that we do not assume that g(t) is real-valued; hence, we are not be able to use any simplification that results from the assumption that g(t) is real valued.

The classical perspective is provided by the Titchmarsh theorem, which works for the function $g\in L^{2}\left(R\right)$.

**Theorem** **1** (see Theorem 1.6.1 in [66] and Theorem 2 in [55])**.** *If a square-integrable function G(ω) fulfills one of the four conditions below, then it fulfills all four of them:*

*(i)*  *The inverse Fourier transform g(t) of G(ω) vanishes for t<0:*
  g(t)=0(t<0).
*(ii)*  *G(v) is, for almost all v, the limit as $u\to 0^{+}$ of an analytic function $\tilde{G}\left(u+jv\right)$ that is holomorphic in the right half-plane and square integrable over any line parallel to the imaginary axis:*
  $$\int_{-\infty }^{\infty }{\left|\tilde{G}\left(u+jv\right)\right|}^{2}dv<C\;\left(u>0\right).$$
*(iii)*  *ℜG and ℑG verify the first Plemelj formula:*
  (147) $$ℜG\left(\omega \right)=-\frac{1}{\pi }{⨏}_{-\infty }^{+\infty }\frac{ℑG(\omega^{\prime })}{\omega^{\prime }-\omega }d\omega^{\prime }.$$
*(iv)*  *ℜG and ℑG verify the second Plemelj formula:*
  (148) $$ℑG\left(\omega \right)=\frac{1}{\pi }{⨏}_{-\infty }^{+\infty }\frac{ℜG(\omega^{\prime })}{\omega^{\prime }-\omega }d\omega^{\prime }.$$

This theorem suggests two different methods of proving the causality, i.e., one requires searching for an appropriate holomorphic extension to the right-half plane, and the other requires to prove the validity of the Kramers-Krönig (K–K) relations (147) and (148).

One should note that for both functions $G_{z}$ and $G_{R}$ given by (96) and (102), respectively, the appropriate holomorphic extensions exist for β∈(0,1), given by

(149) $$G_{z}\left(\sigma +j\omega \right)=G_{z}\left(s\right)={\tilde{F}}_{+}e^{-zc_{\beta }^{-1}s^{\beta }}$$

(150) $$G_{R}\left(\sigma +j\omega \right)=G_{R}\left(s\right)=\frac{1}{R}{\tilde{F}}_{+}e^{-Rc_{\beta }^{-1}s^{\beta }}.$$

Since all the assumptions given in point (ii) of the Titchmarsh Theorem 1 are satisfied, it directly proves the causality.

On the other hand, the case of transfer function with the cylindrical symmetry is different. The function $G_{r}$, given in (109), does not belong to $L^{2}\left(R\right)$ due to the asymptotics of the Bessel function $J_{0}$ given by

(151) $$J_{0}\left(z\right)=\sqrt{\frac{2}{\pi z}}\cos\left(z-\frac{\pi }{4}\right)+e^{\left|ℑz\right|}{O(|z|}^{-3/2})$$

valid for z∈C such that argz∈(−π,π) as |z|→+∞ (see formula 9.2.1 in [67], more details are given in Chapter VII in [57]). The function $G_{r}\left(\omega \right)$ has a natural holomorphic extension to the right half-plane given by

(152) $$G_{r}\left(\sigma +j\omega \right)=G_{r}\left(s\right)={\tilde{F}}_{+}J_{0}\left(js^{\beta }r/c_{\beta }\right).$$

However, since we operate outside $L^{2}\left(R\right)$, we may not directly apply classical methods and we should treat the function $G_{r}\left(\omega \right)$ as a tempered distribution. Fortunately, there are tools that may be applied here as well: we are going to refer to the theorem proved in [68] and rephrased as Theorem 7 and discussed in [55].

**Theorem** **2** (see Theorem 3.8 in [68])**.** *Suppose that $F\in H(C_{+})$ satisfies:*

*(i)*  *for each $\rho_{0}>0$ function F restricted to the set $\left\{s\in C:ℜs>\rho_{0}\right\}$ is of order/type ≤(2,0)*
*(ii)*  *$b={lim sup}_{y\to +\infty }y^{-1}\ln|(F\left(y\right)|$ is finite*
*(iii)*  *there exists R>0 such that for all ρ∈(0,R] the function $F_{\rho }\left(\omega \right)=F\left(\rho +i\omega \right)$ satisfies $F_{\rho }\in S^{\prime }$.*

*Then, there exists such a distribution $f\in D^{\prime }$ that supp(f)⊂[−b,+∞) and F is the Laplace transform of f.*

The condition (i) is equivalent to saying that for any ε>0, $C=C(\varepsilon ,\rho_{0})>0$ exists such that

(153) $$\ln|F\left(s\right)|\leq \ln C+{\varepsilon |s|}^{2}.$$

The function (152) satisfies (due to the estimate (151)) all the assumptions of Theorem 2, so it is also a causal transform.

## 11. Conclusions

In this paper, the extension of TF electrodynamics towards the formulation based on the RS vector is presented. Up to now, the existing formulations of electrodynamics based on the RS vector do not include the energy dissipation; hence, they are limited to a homogeneous and static medium, which is usually a vacuum. The time-domain fractionalization of Maxwell’s equations allows for inclusion of energy dissipation, which is the intrinsic property of media described by FOMs. TF Maxwell’s equations are formulated using the RS vector and their properties are analysed from the point of view of classical electrodynamics, i.e., energy and momentum conservation, reciprocity, causality. Therefore, it is possible to demonstrate that the energy density of the electromagnetic field and media described by FOM, which is spent to create the field and change the momentum of medium, reflects the entire previous history of the process. Hence, the application of FOM allows for description of memory effects in electrodynamics. Afterwards, classical solutions are derived for wave-propagation problems, assuming helical, spherical and cylindrical symmetries of solutions. Furthermore, we demonstrate relations between the TF Schrödinger equation and TF electrodynamics using the proposed formulation based on the RS vector.

## Data Availability

Not applicable.

## References

[1] Baleanu D., Golmankhaneh A.K., Golmankhaneh A.K., Baleanu M.C. (2009). Fractional Electromagnetic Equations Using Fractional Forms. Int. J. Theor. Phys..

[2] Lazo M.J. (2011). Gauge invariant fractional electromagnetic fields. Phys. Lett. A.

[3] Jaradat E.K., Hijjawi R.S., Khalifeh J.M. (2012). Maxwell’s equations and electromagnetic Lagrangian density in fractional form. J. Math. Phys..

[4] Rabei E.M., Al-Jamel A., Widyan H., Baleanu D. (2014). Comment on “Maxwell’s equations and electromagnetic Lagrangian density in fractional form” [J. Math. Phys. 53, 033505 (2012)]. J. Math. Phys..

[5] Ortigueira M.D., Rivero M., Trujillo J.J. (2015). From a generalised Helmholtz decomposition theorem to fractional Maxwell equations. Commun. Nonlinear Sci. Numer. Simul..

[6] Engheta N. (1998). Fractional curl operator in electromagnetics. Microw. Opt. Technol. Lett..

[7] Naqvi Q., Abbas M. (2004). Complex and higher order fractional curl operator in electromagnetics. Opt. Commun..

[8] Tarasov V.E. (2008). Fractional vector calculus and fractional Maxwell’s equations. Ann. Phys..

[9] Bogolyubov A.N., Potapov A.A., Rehviashvili S.S. (2009). An approach to introducing fractional integro-differentiation in classical electrodynamics. Mosc. Univ. Phys. Bull..

[10] Tarasov V.E. (2009). Fractional integro-differential equations for electromagnetic waves in dielectric media. Theor. Math. Phys..

[11] Tarasov V. (2011). Fractional Dynamics: Application of Fractional Calculus to Dynamics of Particles, Fields and Media.

[12] Nasrolahpour H. (2013). A note on fractional electrodynamics. Commun. Nonlinear Sci. Numer. Simul..

[13] Westerlund S. (1991). Dead matter has memory!. Phys. Scr..

[14] Engheta N. (1996). On fractional calculus and fractional multipoles in electromagnetism. IEEE Trans. Antennas Propag..

[15] Engheta N. (1997). On the role of fractional calculus in electromagnetic theory. IEEE Antennas Propag. Mag..

[16] Machado J.T., Jesus I.S., Galhano A., Cunha J.B. (2006). Fractional order electromagnetics. Signal Process..

[17] Veliev E.I., Engheta N. Fractional curl operator in reflection problems. Proceedings of the 10th International Conference on Mathematical Methods in Electromagnetic Theory.

[18] Ivakhnychenko M.V., Veliev E.I., Ahmedov T.M. (2007). Fractional Operators Approach in Electromagnetic Wave Reflection Problems. J. Electromagn. Waves Appl..

[19] Veliev E.I., Ivakhnychenko M.V., Ahmedov T.M. (2008). Fractional boundary conditions in plane waves diffraction on a strip. Prog. Electromagn. Res..

[20] Naqvi S., Naqvi Q., Hussain A. (2006). Modelling of transmission through a chiral slab using fractional curl operator. Opt. Commun..

[21] Weber H. (1901). Die Partiellen Differential-Gleichungen Der Mathematischen Physik: Nach Riemann’s Vorlesungen Bearbeitet von Heinrich Weber.

[22] Silberstein L. (1907). Elektromagnetische Grundgleichungen in bivektorieller Behandlung. Ann. Phys..

[23] Silberstein L. (1907). Nachtrag zur Abhandlung uber “Elektromagnetische Grundgleichungen in bivektorieller Behandlung”. Ann. Phys..

[24] Silberstein L. (1912). LXXVI. Quaternionic form of relativity. Lond. Edinburgh Dublin Philos. Mag. J. Sci..

[25] Bialynicki-Birula I., Bialynicka-Birula Z. (2013). The role of the Riemann–Silberstein vector in classical and quantum theories of electromagnetism. J. Phys. Math. Theor..

[26] Bialynicki-Birula I. (1996). V Photon Wave Function. Prog. Opt..

[27] Bialynicki-Birula I. (1998). Exponential Localization of Photons. Phys. Rev. Lett..

[28] Bialynicki-Birula I., Bialynicka-Birula Z. (2006). Beams of electromagnetic radiation carrying angular momentum: The Riemann-Silberstein vector and the classical-quantum correspondence. Opt. Commun..

[29] Belkovich I.V., Kogan B.L. (2016). Utilization of Riemann-Silberstein Vectors in Electromagnetics. Prog. Electromagn. Res. B.

[30] Belkovich I.V., Kogan B.L. The Riemann-Silberstein vectors theory and vector spherical expansion. Proceedings of the 2017 Progress in Electromagnetics Research Symposium—Spring (PIERS).

[31] Samko S.G., Kilbas A.A., Marichev O.I. (1993). Fractional Integrals and Derivatives: Theory and Applications.

[32] Gulgowski J., Stefanski T.P. On Applications of Fractional Derivatives in Electromagnetic Theory. Proceedings of the 2020 23rd International Conference on Microwave, Radar and Wireless Communications (MIKON).

[33] Gulgowski J., Stefański T.P., Trofimowicz D. (2020). On Applications of Elements Modelled by Fractional Derivatives in Circuit Theory. Energies.

[34] Stefański T.P., Gulgowski J. (2020). Signal propagation in electromagnetic media described by fractional-order models. Commun. Nonlinear Sci. Numer. Simul..

[35] Kilbas A.A., Srivastava H.M., Trujillo J.J. (2006). Theory and Applications of Fractional Differential Equations.

[36] Moreles M.A., Lainez R. (2017). Mathematical modelling of fractional order circuit elements and bioimpedance applications. Commun. Nonlinear Sci. Numer. Simul..

[37] Westerlund S., Ekstam L. (1994). Capacitor theory. IEEE Trans. Dielectr. Electr. Insul..

[38] Curie M.J. (1889). Recherches sur La Conductibilit Des Corps Cristallises. Ann. Chim. Phys..

[39] Naber M. (2004). Time fractional Schrödinger equation. J. Math. Phys..

[40] Achar B.N.N., Yale B.T., Hanneken J.W. (2013). Time Fractional Schrödinger Equation Revisited. Adv. Math. Phys..

[41] Iomin A. (2019). Fractional evolution in quantum mechanics. Chaos Solitons Fractals.

[42] Feynman R.P., Hibbs A.R. (1965). Quantum Mechanics and Path Integrals.

[43] Laskin N. (2000). Fractional quantum mechanics. Phys. Rev. E.

[44] Ryabov Y.E., Puzenko A. (2002). Damped oscillations in view of the fractional oscillator equation. Phys. Rev. B.

[45] Luchko Y., Mainardi F., Povstenko Y. (2013). Propagation speed of the maximum of the fundamental solution to the fractional diffusion–wave equation. Comput. Math. Appl..

[46] Luchko Y. (2013). Fractional wave equation and damped waves. J. Math. Phys..

[47] Luchko Y., Mainardi F. (2014). Cauchy and Signaling Problems for the Time-Fractional Diffusion-Wave Equation. J. Vib. Acoust..

[48] Jackson J.D. (1998). Classical Electrodynamics.

[49] Griffiths D.J. (2013). Introduction to Electrodynamics.

[50] Balazs N.L. (1953). The Energy-Momentum Tensor of the Electromagnetic Field inside Matter. Phys. Rev..

[51] Griffiths D.J. (2012). Resource Letter EM-1: Electromagnetic Momentum. Am. J. Phys..

[52] Gorenflo R., Kilbas A.A., Mainardi F., Rogosin S.V. (2014). Mittag-Leffler Functions, Related Topics and Applications.

[53] Luchko Y., Mainardi F. (2013). Some properties of the fundamental solution to the signalling problem for the fractional diffusion-wave equation. Cent. Eur. J. Phys..

[54] Stefański T.P., Gulgowski J. (2020). Fundamental properties of solutions to fractional-order Maxwell’s equations. J. Electromagn. Waves Appl..

[55] Gulgowski J., Stefański T.P. (2021). Generalization of Kramers-Krönig relations for evaluation of causality in power-law media. Commun. Nonlinear Sci. Numer. Simul..

[56] Gibson W. (2008). The Method of Moments in Electromagnetics.

[57] Watson G.N. (1995). A Treatise on the Theory of Bessel Functions.

[58] Gradshteyn I.S., Ryzhik I.M. (1980). Table of Integrals, Series, and Products.

[59] Stefanski T.P., Gulgowski J. Simulation of Wave Propagation in Media Described by Fractional-Order Models. Proceedings of the 2020 23rd International Microwave and Radar Conference (MIKON).

[60] Stefański T.P., Gulgowski J. (2019). Electromagnetic-based derivation of fractional-order circuit theory. Commun. Nonlinear Sci. Numer. Simul..

[61] Davidovich M.V. (2010). On energy and momentum conservation laws for an electromagnetic field in a medium or at diffraction on a conducting plate. Phys.-Uspekhi.

[62] Campos I., Jimenez J., Lopez-Marino M. (2012). Electromagnetic momentum balance equation and the force density in material media. Rev. Bras. Ensino Sica.

[63] Campos I., Jiménez J.L., Roa-Neri J.A.E. (2016). Radiation force and balance of electromagnetic momentum. Eur. J. Phys..

[64] Campos I., Jiménez J.L., Roa-Neri J.A.E. (2017). Balance Equations of Electromagnetic Angular Momentum. J. Electromagn. Anal. Appl..

[65] Harrington R.F. (2001). Time-Harmonic Electromagnetic Fields.

[66] Nussenzveig H. (1972). Causality and Dispersion Relations.

[67] Abramowitz M. (1974). Handbook of Mathematical Functions, With Formulas, Graphs, and Mathematical Tables.

[68] Carlsson M., Wittsten J. (2017). A note on holomorphic functions and the Fourier-Laplace transform. Math. Scand..

